# Friedreich’s Ataxia and Cesarean Delivery: A Case Report of Epidural Anesthesia With Ropivacaine

**DOI:** 10.7759/cureus.61776

**Published:** 2024-06-06

**Authors:** Polyxeni Theodosopoulou, Marianna Mavromati, Anteia Paraskeva

**Affiliations:** 1 Anesthesia and Pain Medicine, Aretaieion University Hospital, National and Kapodistrian University of Athens, Athens, GRC

**Keywords:** cesarean delivery, epidural anesthesia, neurodegenerative disorders, isobaric ropivacaine, friedreich’s ataxia

## Abstract

Friedreich’s ataxia (FRDA), a rare inherited neurodegenerative disease, presents distinctive complexities in obstetrical anesthesia. Available research about FRDA in obstetrics is extremely limited. In this report, the anesthetic management of a 40-year-old primigravida with FRDA undergoing cesarean delivery is presented. An uneventful cesarean delivery with effective epidural anesthesia with ropivacaine at the L2-L3 intervertebral space was performed in our case. Neither hypotension nor bradycardia was observed, and vital signs remained stable, with no need for administration of vasoactive drugs. After discharge, the parturient reported no change in her neurologic symptoms. Conclusive recommendations are contingent upon more extensive studies. Overall management and the choice to proceed with neuraxial anesthesia in a woman with FRDA should be based on comprehensive consultations in both cardio-obstetrics and pre-anesthetic evaluations.

## Introduction

Friedreich’s ataxia (FRDA) is one of the most common forms of hereditary ataxias, accounting for 50% of all ataxia cases [1]. With a prevalence of 1 in 40,000 people [2], this progressive neurodegenerative disease is characterized by loss of proprioception, tendon reflexes, and sensation; muscle weakness with lack of coordination; difficulty in ambulation; impaired speech; and dysphagia [3,4]. FRDA is an inherited autosomal recessive disorder typically presenting in early adolescence [4,5]. FRDA is caused by a mutation in the frataxin (*FXN*) gene situated on chromosome nine: abnormal *FXN* alleles that contain expansions of GAA trinucleotide repeats present in patients with FRDA [4,5]. This abnormal triplet repeat leads to a reduction in frataxin which is a key protein in mitochondrial function and oxidative phosphorylation [5]. Apart from neurological features, patients with FRDA have other manifestations such as cardiomyopathy, scoliosis, and diabetes mellitus [4]. Loss of proprioception due to posterior column degeneration and loss of tendon reflexes due to loss of sensory ganglia lead to loss of spinal muscle balance and eventually the development of kyphoscoliosis.

Although there have been advances in FRDA drug development, there is no approved therapy that alters the disease progression [4]. Improvement in symptomatic treatment modalities increases the life expectancy of FRDA patients [5]. More women with FRDA are entering childbearing age and have the desire to conceive. This highlights the importance for healthcare providers to be well-acquainted with this specific population [5].

Here, we present the anesthetic management of a 40-year-old woman with FRDA undergoing cesarean delivery. Through a review of previous cases and relevant literature, we also summarize the clinical features and anesthetic considerations in FRDA in obstetrics.

## Case presentation

A 40-year-old primigravida at 39 + 1 weeks of gestation, with a history of FRDA was scheduled for an elective cesarean section. The parturient’s height and weight were 1.60 cm and 98 kg, respectively. The onset of FRDA was at the age of 17. By the age of 31, she was wheelchair dependent.

Upon physical examination, the parturient suffered from severe paraparesis, limb ataxia, and dysarthria. Preoperative imaging of the spinal column was not available to the anesthetic team; however, there were no visual signs of spinal column deformities such as scoliosis. The neurological assessment revealed bilaterally absent tendon reflexes, positive Babinski reflexes, and loss of deep sensibility, with saccadic eye movements, and bilateral nystagmus. The Scale for the Assessment and Rating of Ataxia score was 27.

**Figure 1:**
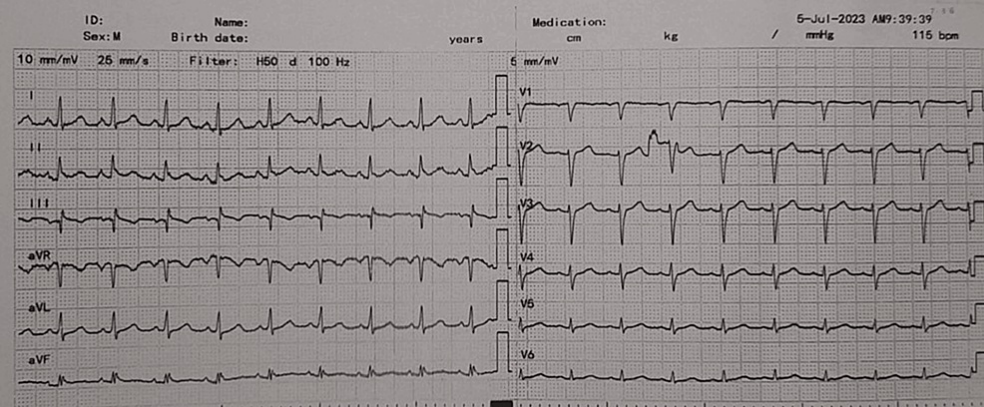
Preoperative electrocardiogram of the patient.

A preoperative electrocardiogram (ECG) recorded a sinus rhythm with an incomplete right bundle branch block (Figure 1). The patient did not complain about any symptoms that could imply cardiac or pulmonary disease. Preoperative echocardiography showed left ventricular diastolic dysfunction with an estimated ejection fraction (LVEF) of 50%. Physical examination and auscultation revealed no signs of cardiovascular or respiratory compromise. She had never undergone surgery in the past and had no known drug allergies.

Laboratory results were within the normal range, allowing the anesthetic team to proceed with the plan of a neuraxial technique for the operation. Written informed consent for undergoing the cesarean section with a lumbar epidural was obtained from the patient.

In the operating room, in addition to the standard monitoring (ECG, pulse oximetry, non-invasive blood pressure), the right radial artery was also catheterized for invasive blood pressure measurements. Intravenous access was established with two 18 G cannulas. The following initial vital signs were noted upon beginning: mean arterial pressure of 103 mmHg, heart rate of 89 beats per minute, and SpO_2_ of 98%.

For performing the neuraxial technique, the patient was positioned in the sitting position. After disinfection and local infiltration of the skin with 5 mL of 2% lidocaine, an 18 G Tuohy needle was inserted at the L2-L3 intervertebral space (Portex epidural Minipack Kit, Smiths Medical). The epidural space was found at 6 cm of depth. Using the loss-of-resistance technique, a polyamide catheter was uneventfully induced and placed at the 11th cm catheter mark. A test dose of 3 mL 2% lidocaine was then administered to confirm the catheter’s proper placement. The patient was then returned to the supine position with a 15-degree left uterine displacement to prevent aorto-caval compression. T4 sensory block was established by 10 mL of ropivacaine 0.75% and 50 µg of fentanyl in aliquots of 2 mL. With the above dosage of local anesthetic, the patient’s motor block was complete (modified bromage scale of 1). Neither hypotension nor bradycardia was observed, and vital signs remained stable, with no need for administration of vasoactive drugs.

A healthy male infant with Apgar scores of 8 and 10 at the first and the fifth minute, respectively, was born by cesarean section. Upon delivery, five units of bolus oxytocin were administered and were followed by a continuous infusion of 17 units of oxytocin in 1,000 mL of normal saline, as per the department’s protocol for cesarean sections. The postpartum period was uneventful and the patient was discharged on the fifth postoperative day. Follow-up six months after discharge revealed no change in symptoms related to FRDA.

## Discussion

FRDA is a multisystemic neurodegenerative disease that affects the neurological, cardiovascular, and endocrine systems [2-4]. Degenerative lesions in FRDA are localized to the proprioceptive system, dentate nucleus of the cerebellum, and corticospinal tracts [1,2]. Cerebellar signs are predominant in FRDA and include ataxia, dysarthria, and nystagmus [5,6] and were present in our case. Patients with FRDA often present with heart disease, scoliosis and pes cavus deformities of the lower extremities, diabetes, and hearing and vision loss [1,3]. It is not uncommon for patients to present with concomitant obstructive sleep apnea, low bone mineral density, urinary dysfunction, and depression [2,4].

Cardiac disease, particularly cardiomyopathy and heart failure, is the most common cause of death for patients with FRDA [4,5]. The cardiomyopathy observed in FRDA results from an increase in mitochondrial proliferation within the myocyte [5]. It is distinctive in its manifestation, presenting as a symmetrical, concentric thickening of the left ventricle without any outflow obstruction [4,5]. Supraventricular arrhythmias and ST-segment and T-wave changes are also observed in individuals with FRDA [5,7]. In our case, the parturient did not present any signs or symptoms of cardiovascular disease and her preoperative ECG and echocardiography were within the normal range, although LVEF appeared reduced. The ejection fraction was maintained at 50%, and the left ventricular wall diameter was in the upper normal limits.

Individuals with FRDA contemplating pregnancy are advised to undergo an ECG and transthoracic echocardiography before initiating conception as an essential step in evaluating the baseline cardiac function [5]. Additionally, follow-up assessments after conception are recommended, ideally at least once per trimester [5]. Brain natriuretic peptide (BNP) should also be measured at baseline and then each trimester. A BNP >100 pg/mL is associated with adverse cardiac events [5]. Although women with FRDA should be encouraged to become mothers, an impaired preconception LVEF warrants caution as pregnancy in such cases is associated with a high rate of maternal and fetal complications [5]. Potential indicators of pregnancy risk in FRDA include an LVEF below 40%, a history of cardiac events or arrhythmias, and the utilization of cardiac medications before the onset of pregnancy [5]. Our patient had undergone cardiac echocardiography once per trimester and was not receiving any cardiac medication.

Women with FRDA who choose to embark on a pregnancy journey should schedule regular meetings with a cardio-obstetric team, ideally once during each trimester, as management of gestation can be challenging and should depend on a multidisciplinary approach [5,8]. Delivery and anesthesia plans should be developed early in the second trimester. As for the post-delivery period, it is crucial that women with FRDA are followed up by a cardiologist, obstetrician, and anesthesiologist [5,7].

Considering disease progression and the possibility of deterioration of symptoms in relation to pregnancy, Friedman et al. performed a retrospective analysis of 31 women examining both the incidence of obstetric complications, maternal and neonatal outcomes, and disease progression [9]. No elevated risks of pre-eclampsia, spontaneous abortion, or preterm birth were observed compared to the general population [9]. The study, on the one hand, demonstrated that obstetric complications were not increased but, on the other hand, the effect of pregnancy and delivery on the disease was inconclusive as both deterioration and amelioration of symptoms were reported equally [9]. Obstetric patients with FDRA need to be informed before conception of the possibility of deterioration of their symptoms, regardless of the delivery methods and the anesthetic technique used. Our anesthetic team had a thorough discussion with our patient beforehand about anesthetic options and postoperative follow-up.

For patients with FRDA, the presence of hypertrophic cardiomyopathy, a high risk of aspiration, and restrictive lung physiology due to thoracic scoliosis make neuraxial analgesia the recommended choice in obstetrics [5,7]. Regarding general anesthesia, both volatile and intravenous agents have been used successfully on non-obstetric patients with FRDA [10]. The use of succinylcholine is contraindicated in such individuals, given the heightened risk of hyperkalemic cardiac arrest [5,6,11]. For the rest of the non-depolarizing muscle relaxants, studies suggest that the responses of the FRDA patient population are similar to their non-FRDA counterparts [12,13].

According to the literature, the preferred anesthesia choice for parturients with FRDA undergoing a cesarean section, as well as for the non-FRDA obstetric population is neuraxial anesthesia [6,8,9,14,15].

Kubal et al. reported a spinal technique with a 22 G needle, where they injected 6 mg of tetracaine intrathecally in a parturient with FRDA, severe scoliosis, and restrictive lung disease [6]. Harmon et al also preferred a spinal technique with a 25 G needle, through which they injected 12 mg of 0.5% hyperbaric bupivacaine along with 25 µg of fentanyl in a parturient with slight lumbar scoliosis and no pulmonary or cardiovascular compromise [14]. In both case reports, adequate T4 sensory level was reached with no adverse events intraoperatively. In both cases, the presence of scoliosis guided the anesthetic teams toward a spinal technique instead of an epidural to avoid problems with local anesthetic spread in the vertebral column that could result in a “patchy” sensory block [6,14].

The lack of severe scoliosis in our patient and the anesthetic team’s wish to achieve adequate sensory block slowly and incrementally to avoid any hemodynamic instability made the lumbar epidural a first-line choice in our case. As FRDA is associated with autonomic dysfunction [16], even though the cardiologic examination of our parturient was within normal values, we wanted to avoid abrupt changes in the patient’s preload and afterload with a spinal technique [7]. Epidural analgesia and anesthesia in obstetrics can be used successfully and are one of the first-line choices in parturients with cardiac disease [7]. Additionally, it has been widely reported as a safe anesthetic choice for parturients with spinal disease and deformities [17]. As ropivacaine is proven to be equipotent with bupivacaine but with a better differential block and a higher cardiac safety profile [18], it was our drug of choice.

To our knowledge, our case report is only the second in the literature that reports the use of ropivacaine in a neuraxial technique for patients with FRDA. Hanusch et al. chose epidural anesthesia in a 25-year-old woman with FRDA and a history of progressive scoliosis, for which dorsal stabilization surgery was performed from T5 to L1 [8]. They considered that epidural anesthesia was more suitable due to its better control of local anesthetics and the advantage of postoperative pain management [8]. A patient-controlled epidural analgesia pump with a ropivacaine-sufentanil mixture was connected to the epidural catheter for three postoperative days with satisfactory outcomes [8]. In both our case and the one by Hanusch et al., the dose of local anesthetic needed to reach adequate sensory block was significantly reduced (10 mL of 0.75% ropivacaine in our case, 8 mL of 0.75% ropivacaine by Hanusch et al.) [8].

The combined spinal-epidural technique was reported in one case report by Wyatt et al. regarding a 23-year-old parturient with FRDA and cardiomyopathy presenting for a vaginal delivery [15]. The parturient had also undergone thoracic spinal fusion as a treatment for scoliosis [15]. Adequate analgesia was provided by 2.5 mg of bupivacaine and 25 µg of fentanyl, administered intrathecally. However, the later use of the in situ epidural catheter with top-ups of bupivacaine and fentanyl was ineffective, probably due to the severely altered spinal anatomy of the patient [15].

## Conclusions

There is limited data and a small number of case reports on neuraxial anesthesia in parturients with FRDA. Nevertheless, no clear indication of adverse events or relapses following obstetrical neuraxial anesthesia seems to exist. In correlation to the existing literature, our six-month follow-up of the parturient failed to reveal any alteration to the clinical symptomatology related to FRDA.

This case report contributes to the existing literature by reinforcing the current view that women with FRDA can achieve a successful pregnancy and delivery with neuraxial anesthesia. The decision of conception and the choice of delivery method and anesthetic technique for delivery should always be made after thorough cardio-obstetrics and pre-anesthetic counseling.
